# Norcyperone, a Novel Skeleton Norsesquiterpene from *Cyperus rotundus* L

**DOI:** 10.3390/molecules13102474

**Published:** 2008-10-10

**Authors:** Yan Xu, Hong-Wu Zhang, Chang-Yuan Yu, Yang Lu, Ying Chang, Zhong-Mei Zou

**Affiliations:** 1 Institute of Medicinal Plant Development, Chinese Academy of Medical Sciences and Peking Union Medical College, Beijing 100193, P. R. China; E-mail: xuyantea@163.com (Y. X.), hongwuzhang77@hotmail.com (H-W. Z.); 2 College of Life Science and Technology, Beijing University of Chemical Technology, Beijing 100029, P. R. China; E-mail: yucy@mail.buct.edu.cn (C-Y. Y.); 3 Institute of Materia Medica, Chinese Academy of Medical Sciences and Peking Union Medical College, Beijing 100050, P. R. China; E-mail: luy@imm.ac.cn (Y. L.),

**Keywords:** *Cyperus rotundus* L., norsesquiterpene, norcyperone, bicyclo[5.3.1]undecane-3-one

## Abstract

A novel norsesquiterpene, named norcyperone (**1**), and three known compounds: (-)-clovane-2,9-diol (**2**), rosenonolactone (**3**), and 5*α*,8*α*-epidioxy-(20*S*,22*E*,24*R*)-ergosta-6,22-dien-3*β*-ol (**4**) were isolated from the rhizomes of *Cyperus rotundus* L. The structure of **1** was elucidated as 8,11,11-trimethylbicyclo[5.3.1]undecane-5*α*, 8*α*-epoxy-3-one on the basis of extensive spectroscopic analyses, including 1D- and 2D-NMR, MS experiments, and single-crystal X-ray diffraction. This is the first report of a 8,11,11-trimethyl- bicyclo[5.3.1]undecane-3-one type norsesquiterpene with a tetrahydrofuran ring at C-5 and C-8.

## Introduction

The rhizomes of *Cyperus rotundus* L. have been used in traditional Chinese medicine as an estrogenic and anti-inflammatory agent for the treatment of women's diseases and also used for treatment of stomach ache and bowel disorders [1]. The extract of the rhizomes of *C. rotundus* L. showed anti-diabetic activity [2], acetylcholinesterase inhibitory activity [3] and antidiarrhoeal activity [4], as well as inhibition of nitric oxide and superoxide production [5]. Previous phytochemical studies on *C. rotundus* L. have led to the identification of more than 60 sesquiterpenes with a diversity of skeletons [6, 7] besides flavonoids, furochromones, triterpenes and sterols [8, 9]. In the course of our phytochemical investigation of this species, a novel norsesquiterpene **1**, named norcyperone, together with three known compounds: (-)-clovane-2,9-diol (**2**), rosenonolactone (**3**), and 5*α*,8*α*-epidioxy-(20*S*, 22*E*,24*R*)-ergosta-6,22-dien-3*β*-ol (**4**) were isolated from the rhizomes of *C. rotundus* L. Herein we report the isolation and structural elucidation of these compounds.

## Results and Discussion

**Figure 1:**
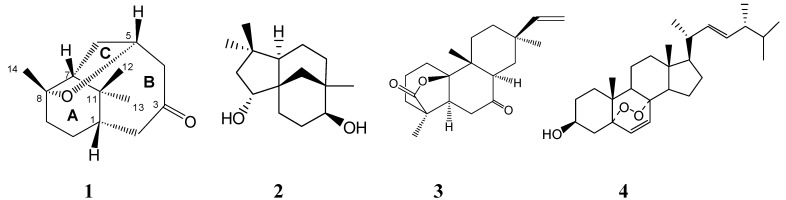
Structures of compounds **1-4**.

The 95% EtOH extract of dried and powdered rhizomes of *C. rotundus* L. was suspended in H_2_O and then extracted successively with petroleum ether, CH_2_Cl_2_, EtOAc and n-BuOH,. The CH_2_Cl_2_-soluble fraction was separated by repeated column chromatography to afford a novel norsesquiterpene, norcyperone (**1**) and three known compounds **2**-**4** (Figure 1).Compounds **2**, **3** and **4** were identified as (-)-clovane-2,9-diol (**2**) [10], rosenonolactone (**3**) [11], and 5*α*,8*α*-epidioxy-(20*S*,22*E*,24*R*)-ergosta-6,22-dien-3*β*-ol (**4**) [12,13] by comparing their physical and spectroscopic data with those reported in the literatures.

**Figure 2:**
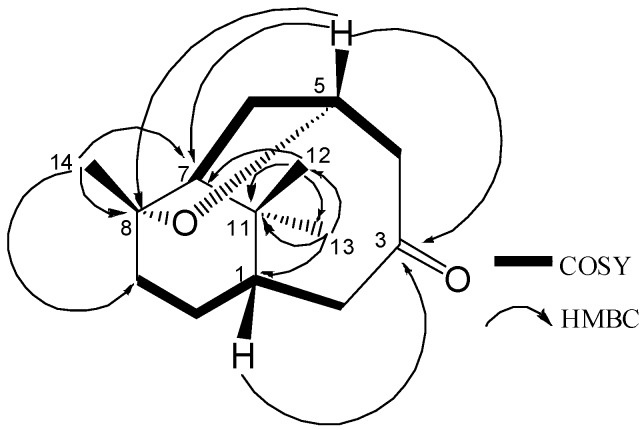
Key HMBC and ^1^H-^1^H COSY correlations of **1.**

Compound **1** was obtained as colourless needles in CH_2_Cl_2_, mp 146-148°C, [*α*]^20^_D__－_54.3° (*c*=0.09, CH_2_Cl_2_). Its IR spectrum showed a strong carbonyl group absorption at 1693 cm^-1^. The molecular formula of **1 ** was assigned as C_14_H_22_O_2_ by HR-ESI-MS (*m/z* 245.1514 [M+Na]^+^, calcd. 245.1512 [M+Na]^+^), with four degrees of unsaturation. The complete assignment of all protons and carbons for** 1** was made by ^1^H-NMR, ^13^C-NMR, ^1^H-^1^H COSY, HMQC and HMBC (Table 1). Its ^13^C-NMR spectrum gave 14 carbon signals including two oxygenated ones at *δ*_C_ 83.8 and 72.2, respectively, and a ketone carbon signal at at *δ*_C_ 211.3. The remaining carbon signals were three methyl groups, five methylene units, two methine units and one quaternary carbon. The ^1^H-NMR spectrum of **1** exhibited three singlet methyls at *δ*_H_ 1.11, 1.14 and 1.17, as well as a proton signal of oxygen-bearing carbon at *δ*_H_ 4.39. The ^1^H-^1^H COSY correlations between H-1/H-2a, 2b, H-1/ H-10a, 10b, H-9b/H-10b, H-5/H-4a, 4b, H-6a/H-5, 7, and H-6b/H-5, 7 revealed partial structures shown by bold lines in Figure 2.

The location of the ketone carbonyl group was established at C-3 by the HMBC correlations of the carbonyl carbon signal (*δ*_C _211.3) with the resonances of H-1, H-2, H-4 and H-5, while the protons of the methyl singlet at *δ*_H_ 1.11 and 1.14 gave HMBC correlations to *δ*_C _38.6 (C-1), *δ*_C _50.6 (C-7), and *δ*_C _35.1(C-11) suggesting the two geminal methyls linked at C-11. In turn, the HMBC spectrum exhibited correlations of the methyl protons (*δ*_H_ 1.17) with *δ*_C _50.6 (C-7), *δ*_C _83.8 (C-8), and *δ*_C _30.3 (C-9), in good agreement with a methyl group linked at C-8. The presence of a tetrahydrofuran ring was deduced by the molecular formula as well as the HMBC correlation between the proton of oxygen-bearing carbon at *δ*_H_ 4.39 (H-5) and C-8 (Figure 2). All above data indicated that compound **1** was a 8, 11, 11-trimethyl- bicyclo[5.3.1]undecane-3-one type norsesquiterpene with a tetrahydrofuran ring at C-5 and C-8. 

**Figure 3:**
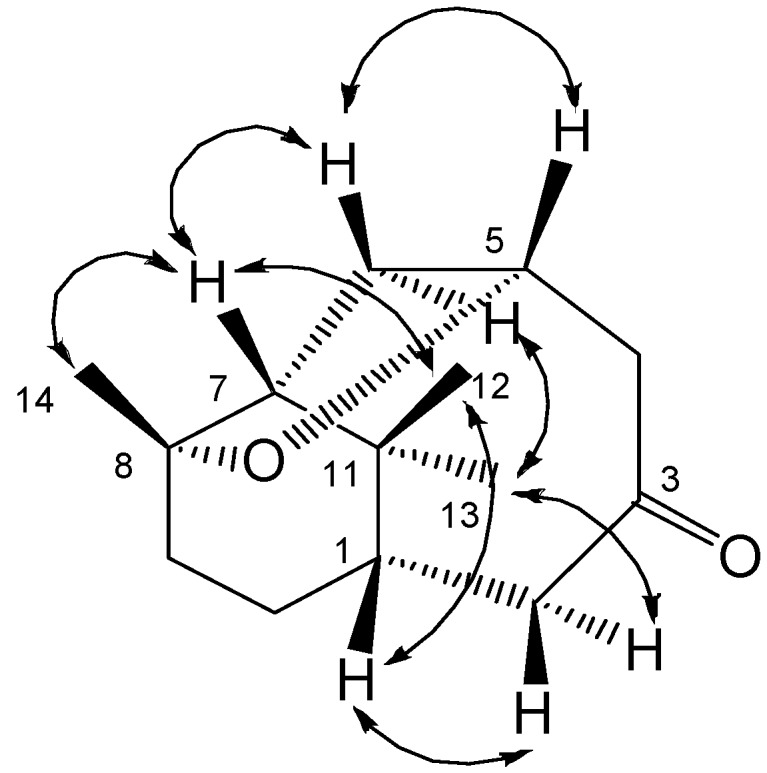
Key NOESY correlations of **1.**

**Figure 4:**
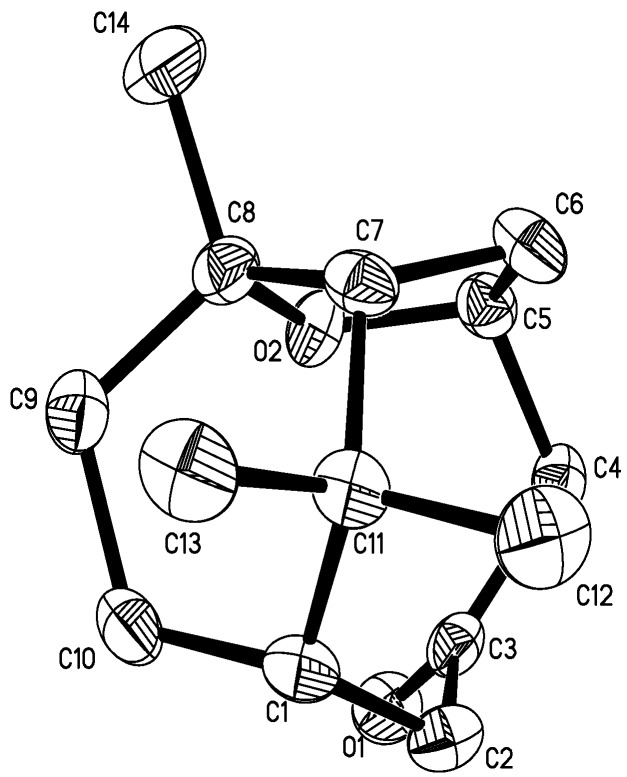
X-ray structure of compound **1.**

The relative stereochemistry of **1** was determined on the basis of a NOESY spectrum. The NOESY correlations between H-1/CH_3_-12, CH_3_-12/ H-7, H-7/CH_3_-14, H-5/H-6a, H-6a/H-7 suggested that all these atoms were in *β*-orientation (Figure 3), which further confirmed by single-crystal X-ray diffraction (Figure 4). The crystal structure showed a six-numbered ring *A* in ‘twist boat’ conformation, an eight-numbered ring *B* in ‘boat’ conformation, and a five-numbered tetrahydrofuran ring *C* in an ‘envelope’ conformation, in which rings *A* and *C* are cis-fused. Thus, the structure of **1** was elucidated as 8,11,11-trimethylbicyclo[5.3.1]undecane-5*α*,8*α*-epoxy-3-one (1*β*-H,7*β*-H), named norcyperone.

**Table 1:** The NMR data of compound **1**^a^.

Position	^1^H-NMR	^13^C-NMR	HMBC (H→C)
1	1.88 (1H, m)	38.6	C-3, 7, 9, 12, 13
2a	2.86 (1H, dd, 12.6, 1.8)	47.9	C-1, 3, 10
2b	2.34 (1H, ddd, 12.6, 7.8, 1.2)		C-1, 3, 4, 10, 11
3	-	211.3	
4a	2.88 (1H, ddd, 13.2,7.8, 1.8)	52.6	C-2, 3, 5, 6
4b	2.62 (1H, dd, 13.2, 3.0)		C-3, 5, 6
5	4.39 (1H, ddt, 10.2, 7.2, 1.8)	72.2	C-3, 7, 8
6a	2.64 (1H, dd, 14.4, 10.2)	35.4	C-4, 5, 7, 11
6b	2.18 (1H, br d, 14.4)		C-4, 7, 8, 11
7	1.77 (1H, d, 10.2)	50.6	C-1, 5, 12, 14
8	-	83.8	
9a	1.85 (1H, m)	30.3	C-1, 7, 8, 10
9b	1.79 (1H, m)		C-8, 10
10a	1.94 (1H, m)	19.6	C-1, 2, 8, 9
10b	1.90 (1H, m)		C-1, 2, 8, 9
11	-	35.1	
12	1.14 (3H, s)	34.3	C-1, 7, 11, 13
13	1.11 (3H, s)	28.4	C-1, 7, 11, 12
14	1.17 (3H, s)	30.1	C-7, 8, 9

^a 1^H-NMR (600 MHz), ^13^C-NMR (150 MHz) spectra of compound **1 **were measured in CDCl_3_ on a Varian Inova-600 MHz spectrometer, *δ* in ppm, *J* in Hz.

## Conclusions

A novel skeleton norsesquiterpene, norcyperone, together with three known compounds **2**-**4** were isolated from the rhizomes of *C. rotundus* L. Although a 8,11,11-trimethylbicyclo[5.3.1]undecane has been reported as a synthetic intermediate derived in the course of synthesis of the taxane-AB-fragment with a spiro-cyclopropyl group [14], this is the first report of a 8,11,11-trimethyl-bicyclo[5.3.1]undecane-3-one type norsesquiterpene with a tetrahydrofuran ring at C-5 and C-8. Compounds **2**-**4 **were isolated from the genus *Cyperus* for the first time. These findings enrich our knowledge of the chemical constituents of *C. rotundus* L.

## Experimental

### General

Melting points were determined on Fisher-Johns Melting Point Apparatus and are uncorrected. IR spectra were measured with a Bruker IFS-55 infrared spectrometer (KBr). Optical rotations were recorded on a Perkin-Elmer 241 automatic recording spectropolarimeter. EI mass spectra were recorded on a Zabspec E mass spectrometer. HR-ESI-MS was taken on a LTQ Orbitrap^TM^ Mass spectrometer. Column chromatography was performed over Silica gel (200-300 mesh) (Qingdao Marine Chemical Factory) and Sephadex LH-20 (Pharmacia). Nuclear magnetic resonance (NMR) spectra were measured on a Varian Inova-600 spectrometer operating at 600 MHz (^1^H) and 150 MHz (^13^C), respectively.

### Plant material

The dried rhizomes of *C. rotundus* L. were collected from Dabieshan Mountains of Anhui Province, P.R. China in Sep. 2006. A voucher specimen (No: 20060825) was authenticated by Prof. Yulin Lin and deposited at the Herbarium of the Institute of Medicinal Plant Development, Chinese Academy of Medical Sciences and Peking Union Medical College.

### Extraction and isolation

The dried and powdered rhizomes of *C. rotundus* L. (15 kg) were extracted with 95% EtOH three times under reflux, and the solvent was evaporated *in vacuo*. The residues were partitioned in H_2_O and extracted successively with petroleum ether, CH_2_Cl_2,_ EtOAc and n-BuOH. The CH_2_Cl_2_ extract (158 g) was submitted to column chromatography on silica gel eluting with petroleum ester/EtOAc (50:1 to 0:1) to afford eight fractions (F_1_-F_8_). Fraction F_2_ (22 g) was further separated by silica gel column chromatography eluting with petroleum ether/acetone (20:1 to 2:1) to give 8 fractions (F_2a_-F_2h_). Fraction F_2d_ was purified by silica gel column chromatography eluting with petroleum ether/acetone (10:1) and Sephadex LH-20 eluted with CH_2_Cl_2_/MeOH (1:1) to obtain **3 **(14 mg). Fractions F_2f_ was purified by silica gel column chromatography eluting with petroleum ester/acetone (4:1) to obtain **4 **(24 mg). F_5_ (18 g) was further chromatographed on silica gel eluted with petroleum ester/acetone (6:1 to 1:1) to give 10 fractions F_5a_-F_5j_. Fraction F_5b_ was purified by silica gel eluted with petroleum ether /acetone (5:1) and Sephadex LH-20 eluted with CH_2_Cl_2_/MeOH (1:1) to obtain **1 **(16 mg). Fraction F_5e_ was purified by Sephadex LH-20 eluting with CH_2_Cl_2_/MeOH (1:1) to obtain **2 **(10 mg).

*Norcyperone* (**1**): colorless needles; mp 146-148°C; [*α*]^20^_D__－_54.3° (c=0.09, CH_2_Cl_2_); HR-ESI-MS *m/z* 245.1514 [M+Na]^+^, calcd. 245.1512 [M+Na]^+^. IR υ_max_ cm^-1^: 2968, 2935, 2881, 1693, 1371, 1066; ^1^H-NMR and^ 13C^-NMR data: see Table 1.

*(-)-Clovane-2, 9-diol* (**2**): colourless needles; mp 162-164°C; EI-MS *m/z*: 238(M), 220, 182, 164, 135, 107, 93, 41. ^1^H-NMR (CD_3_COCD_3_) *δ*_H:_ 0.85 (3H, *s*, CH_3_-13), 0.91 (3H, *s*, CH_3_-15), 1.01 (3H, *s*, CH_3_-14), 3.22 (1H, *m*, H-9), 3.72 (1H, m, H-2), 1.43 (1H, *m*, H-5); ^13^C-NMR (CD_3_COCD_3 _) *δ*_C_: 45.4 (C-1), 80.8 (C-2), 48.5 (C-3), 37.6 (C-4), 51.5 (C-5), 34.1 (C-6), 21.5 (C-7), 35.4 (C-8), 74.9 (C-9), 27.2 (C-10), 27.5 (C-11), 36.4 (C-12), 25.8 (C-13), 31.9 (C-14), 29.2 (C-15).

*Rosenonolactone* (**3**): white needles, mp 224-226°C; EI-MS *m/z*: 316(M), 301, 220, 163, 108, 93, 41. ^1^H-NMR (CDC1_3_) *δ*_H:_ 0.95 (3H, *s*, CH_3_-20), 0.97 (3H, *s*, CH_3_-17), 1.13 (3H, *s*, CH_3_-18), 4.92 (1H, *dd*, *J*=10.8, 0.6 Hz, H-16a), 4.99 (1H, *dd*, *J*=17.4, 1.2 Hz, H-16b), 5.83 (1H, *dd*, *J*=17.4, 10.8 Hz, H-15); ^13^C-NMR (CDCl_3_) *δ*_C_: 30.3 (C-1), 19.8 (C-2), 35.5 (C-3), 47.3 (C-4), 50.9 (C-5), 35.8 (C-6), 210.2 (C-7), 47.4 (C-8), 38.9 (C-9), 86.9 (C-10), 30.8 (C-11), 31.4 (C-12), 35.1 (C-13), 31.7 (C-14), 149.6 (C-15), 109.9 (C-16), 21.9 (C-17), 16.9 (C-18), 179.2 (C-19), 16.8 (C-20).

*5α,8α-Epidioxy-(20S,22E,24R)-ergosta-6,22-dien-3β-ol* (**4**): colourless needles; mp 155-157°C; EI-MS *m/z*: 428 (M), 396, 251, 152, 107, 93, 81, 69; ^1^H-NMR (CDCl_3_) *δ*_H:_ 6.50 (1H, *d*, *J*=8.5 Hz, H-6), 6.24 (1H, *d*, *J*=8.5 Hz, H-7), 5.21 (1H, *dd*, *J*=15.5, 8.0 Hz, H-23), 5.14 (1H, *dd*, *J*=15.5, 8.0 Hz, H-22), 3.97 (1H, *m*, H-3), 1.00 (3H, *d*, *J*=7.0 Hz, H-21), 0.91 (3H, *d*, *J*=7.0 Hz, H-28), 0.88 (3H, *s*, H-19), 0.84 (3H, *s*, H-18), 0.82 (3H, *d*, *J*=5.0 Hz, H-26), 0.81(3H, *d*, *J*=3.0 Hz, H-27); ^13^C-NMR (CDCl_3_)* δ*_C_: 34.7 (C-1), 30.1 (C-2), 66.5 (C-3), 36.9 (C-4), 82.1 (C-5), 135.4 (C-6), 130.8 (C-7), 79.4 (C-8), 51.1 (C-9), 36.8 (C-10), 23.4 (C-11), 39.4 (C-12), 44.6 (C-13), 51.7 (C-14), 20.6 (C-15), 28.6 (C-16), 56.2 (C-17), 13.0 (C-18), 18.2 (C-19), 39.9 (C-20), 20.9 (C-21), 135.2 (C-22), 132.3 (C-23), 42.8 (C-24), 33.1 (C-25), 19.9 (C-26), 19.6 (C-27), 17.6 (C-28).

### Crystallographic Data

Crystallographic data for **1**: formula: C_14_H_22_O_2_; molecular weight: 222.32; monoclinic, space group *P*2_1_2_1_2_1_, *a* = 7.450 (15), *b* = 8.691(17), *c* = 18.981 (4) Å; *V* = 1228.8(4) Å^3^, *Z* = 4, *d* =1.202 g/cm^3^; crystal dimensions, 0.15×0.15×0.30 mm. The measurements were performed on a *MAC DIP-2030K* diffractometer with a graphite monochromator (*ω*-2*θ* scans, 2*θ* max = 50.0°), Mo*K*a radiation. The total number of independent and observed reflections was 2423 (|*F*|^2^ *≥*2σ |*F*|^2^). The crystal structure was solved by direct methods using SHELXS-97, expanded using difference Fourier techniques, and refined with NOMCSDP using full-matrix least-squares calculations. Final indices: *R1* = 0.0395, *wR2* = 0.0991, *S* = 1.038. Crystallographic data for **1** has been deposited at the Cambridge Crystallographic Data Center. CCDC 694682 contains the supplementary crystallographic data for this paper. These data can be obtained free of charge via www.ccdc.cam.ac.uk/conts/retrieving.html (or from the CCDC, 12 Union Road, Cambridge CB2 1EZ, UK; fax: +44 1223 336033; e-mail: deposit@ccdc.cam.ac.uk).
